# Avança Saúde SP: driving the digital transformation of primary healthcare in São Paulo

**DOI:** 10.1093/oodh/oqaf026

**Published:** 2025-10-06

**Authors:** Cíntia Borges Almeida da Fonseca, Leonardo G Shibata, Márcia Gomes Rocha, Jennifer A Nelson

**Affiliations:** Pontifícia Universidade Católica do Rio de Janeiro, 225 Marquês de São Vicente Street, Gávea, Rio de Janeiro, RJ, Brazil; Digital Solutions, Social Protection and Health Division, Inter-American Development Bank, Alameda Santos, 2300, 2º andar, Cerqueira Cesar, São Paulo, SP, 01418-200, Brazil; Health Digital Solutions, Social Protection and Health Division, Inter-American Development Bank, Alameda Santos, 2300, 2º andar, Cerqueira Cesar, São Paulo, SP, 01418-200, Brazil; Health Digital Solutions, Social Protection and Health Division, Inter-American Development Bank, 1300 New York Avenue, N.W., Washington, D.C. 20577, USA

**Keywords:** electronic health record (EHR), interoperability, digital health, health system management, telemedicine, primary healthcare (PHC) resilience, Brazil

## Abstract

This study examines how digital tools can redesign primary healthcare systems, focusing on the digital transformation activities implemented by the Avança Saúde SP program in São Paulo, Brazil. With investments of US$200 million, the 5-year program (2019–2024) aimed to expand coverage and improve primary healthcare for the São Paulo population through investments in technology, management and infrastructure. Based on interviews with healthcare professionals and public officials, this case outlines the initiative’s use of integrated systems for administrative and care services, telecare and risk management. The findings suggest that these actions have contributed to a more resilient, integrated and data-driven healthcare network in São Paulo. The program’s combination of strategic leadership, interoperable digital platforms and integrated management systems demonstrates a potentially replicable model for strengthening primary healthcare resilience, offering valuable insights for other cities seeking to enhance health system preparedness, equity and efficiency.

## INTRODUCTION

São Paulo is Brazil’s most populous city with >11 million inhabitants and the leading financial center, contributing to 9.2% of the country’s gross domestic product [1]. Despite improvements in longevity, education and income, and moving from a ‘high’ to the ‘very high’ ranking on human development index on the period 2000–2010 [2], the city still exhibits a wide range of socioeconomic disparities that led to health inequities ([3], p. 2). The pandemic crisis, along with emerging and pre-existing public health challenges such as climate change, has further underscored the imperative of employing a primary healthcare (PHC) approach to attain universal access to health and universal health coverage [4].

The city’s sociodemographic complexity amplifies the challenge of implementing a primary healthcare model capable of meeting the multiple needs and demands of serving and caring for the population based on the principles of universality, integrality and equality advocated by the Brazilian Unified Health System (SUS). Uneven economic development has failed to eliminate the pockets of structural poverty that are spread throughout the municipality and are reflected in illness rates and types. Moreover, the city’s population is not only aging rapidly, as an expected 30% is projected to be >60 years old by 2050, from 14.7% in 2018 [5], but this aging is geographically uneven within the city.

The municipal health department has the dual challenge of addressing the increased number of elderly people in the city and reducing inequalities. This includes chronic non-communicable diseases, which represent the largest burden of disease in the municipality and a large share of hospitalization ([6], p. 18). The analysis of the spatial distribution of major causes of mortality in São Paulo reveals that ‘poorer economic conditions correlate with a higher risk of non-communicable diseases (NCDs)’ ([7], p. 8), and the COVID-19 reinforced the health inequality caused by social vulnerability conditions ([7], p. 7). Furthermore, the same study shows that the cardiovascular risk of São Paulo residents increases with the precariousness of the urban indicators of their residential area ([7], p. 15).

The city’s complexity generates a mismatch between supply and demand for health services in different regions of the urban conglomerate. This is aggravated by the increase in the number of SUS users, which is already the exclusive provider for 7 out of 10 Brazilians nationally [8]. Consequently, in the early 2010s the municipality faced significant deficits in primary care units, especially in poor regions where care units lack basic infrastructure and lacked adequate health workforce and real-time data management systems for supplies and medication, leading to emergency purchases and expired products. Furthermore, there was no unified data system for patient care, with fragmented paper records and >20 disjointed electronic medical record systems across the city.

In response, the São Paulo Municipal Health Department, supported by the Inter-American Development Bank, launched Avança Saúde SP (2019–2024)—a US$200 million initiative to improve the resilience and equity of the primary care system. The program focused on expanding access and modernizing care through investments in technology, digital integration, telecare and infrastructure to expand coverage and improve the primary healthcare offered to the population. The program aligns with the Pan American Health Organization's (PAHO) Roadmap for the Digital Transformation of the Health Sector in the Region of the Americas [9], which sets principles such as universal connectivity, inclusive digital solutions, interoperable systems, data security and integration with public health structures, as well as with the PAHO–IDB collaboration under the IS4H framework [10], principles reflected in São Paulo’s Avança Saúde SP.

This case study examines Avança Saúde SP to show how digital transformation can support the redesign of primary care, strengthen health system resilience, and improve service delivery and decision-making through better data use in both clinical and administrative practices.

## MATERIALS AND METHODS

This case study is based on 13 semi-structured interviews conducted in 2023 with 8 key stakeholders from the São Paulo Municipal Health Department directly involved in implementing Avança Saúde SP. Participants were purposefully selected for their roles in clinical data management, telecare coordination, risk classification and administrative systems. Three participants were interviewed more than once: the general project coordinator (three interviews), the IT sector coordinator and technology assistant (two joint interviews) and the technology assistant (one additional individual interview).

Interviews were audio-recorded, transcribed and analyzed thematically, with codes focused on digital transformation, system integration, management processes and operational outcomes. To triangulate findings, we analyzed program documents and secondary data provided by SMS (São Paulo Municipal Health Department) and CEINFO Health Data Bulletin, as well as reports from international organizations (PAHO, IDB, World Bank) [4, 11, 12] and scientific literature on digital health. Conducted during the program’s final implementation phase, the interviews served as the primary empirical basis, providing detailed insights into program dynamics, challenges and lessons learned, which were integrated with documentary evidence to inform the Results and Discussion sections.

## RESULTS

This section highlights key investments in primary care aimed at improving service delivery, patient engagement and system resilience. It focuses on the development and implementation of E-Saúde SP as an integrated clinical data repository and app, the expansion of telecare services to reach patients remotely and the adoption of the Manchester risk classification protocol to standardize urgency assessment across the city’s health network. These innovations collectively improve access, efficiency and patient self-management.

### E-Saúde SP: integrated platform and application

The program’s investments in technology and digital health began with the development of E-Saúde SP, a clinical data repository capable of connecting multiple systems and institutions in line with the Ministry of Health’s interoperability recommendations and anticipating requirements of the Digital Health Strategy for Brazil 2020–28 (MDS, 2020) [13, 14]. Before this implementation, the Secretariat lacked a unified system for data and processes, limiting its ability to obtain an organized view of citizen health. With E-Saúde SP, information is structured by target populations (e.g. diabetics, pregnant women), enabling strategic decision-making based on data analysis.

E-Saúde SP integrates various systems and medical records from the São Paulo health network, consolidating data from primary, specialized and hospital care, as well as laboratories. Rather than imposing a new system, Avança Saúde SP invested in integrating legacy data from existing technological bases. According to representatives of the São Paulo Health Department responsible for implementing the Avança Saúde SP project, by 2023 the repository had consolidated >26 million clinical histories, covering a metropolis of >11 million residents, across a network of >1000 facilities and supported by ~106 000 professionals [6].

The E-Saúde SP app provides patients with access to their medical history, test results and vaccination records. Before the application, patients from remote areas seeking specialized care had to restart their treatment at a basic health unit (UBS) without access to previous clinical data. The lack of integrated data compromised care quality and diagnosis accuracy, and led to unnecessary consultations and examinations.

Launched during the COVID-19 pandemic, the E-Saúde SP app enabled vaccine scheduling, teletriage and electronic prescriptions, supporting >93 000 remote consultations in its initial rollout. By mid-2023, the app had registered 15.6 million logins and 3.1 million active users, making it São Paulo’s most downloaded municipal application. These figures gain significance when contextualized against the scale of the municipal network, which carried out ~28 million consultations in 2022 alone, according to data shared by São Paulo Health Department [15].

The platform’s functionalities expanded in sequence, reflecting both prior investments and pandemic-driven needs. Tools such as the Glycemic Monitoring Program (2005), Central Mãe Paulistana (2006) and Agenda Fácil (2017) were integrated into E-Saúde SP in 2020, consolidating previously fragmented digital services. In June 2020, during the height of the pandemic, the Secretariat launched Minha Saúde, the Central Covid and the E-Saúde SP app itself, providing real-time solutions for triage, scheduling and remote monitoring. Later, in August 2021, the Vaccine Passport was introduced, replacing the paper vaccination card and strengthening the integrated ecosystem. This sequencing illustrates how Avança Saúde SP balanced the incorporation of legacy programs with the accelerated rollout of new tools in response to the public health emergency.

Beyond expanding access, E-Saúde SP also supports self-care and care coordination. The Central Mãe Paulistana Digital alone facilitated >108 000 remote maternal and childcare consultations in 2 years, while Agenda Fácil helped users manage appointments and access their full care history.

### Telecare

São Paulo is a city marked by significant social inequities, which directly affect access to primary health care. Transport barriers and a shortage of healthcare professionals in remote areas often delay diagnoses and treatments, particularly for patients with limited mobility. While telemedicine offers a potential solution, low digital literacy and poor internet access can also limit its effectiveness. To address these challenges, the city launched a teleservice program providing remote consultations through hybrid offices, apps and home visits.

The initiative began with the Central Covid app during the pandemic, later expanding into Pronto Saúde, which enabled virtual consultations with general practitioners. From September 2022 to 2023, the program recorded >90 000 COVID-related and 3500 general teleconsultations. It also allowed patients to book appointments and access specialized care and diagnostic examinations.

Home teleservices provide an alternative for patients unable to travel, with family health teams equipped with tablets and internet access to support those with low digital literacy or limited connectivity*.* This approach improved time, resources and staff management by allowing healthcare professionals to reach patients at home, enhancing convenience for patients and optimizing doctors’ schedules.

Among teleservices, the model with the highest user acceptance is the hybrid office, in which specialists connect remotely with patients who are at a health unit with access to connectivity and the technological equipment provided by the municipality ensuring that patients with limited digital skills can complete consultations without navigating technology on their own. The hybrid office is already available at 57 health establishments in the municipality, adding agility and quality to the care of low-priority patients who arrive at municipal units. Three hybrid offices have been installed in emergency care units (UPAs) and the rest in basic health units (UBSs).

From the project’s launch in October 2022 to September 2023, >28 000 consultations were conducted in hybrid offices across São Paulo, and users reported high satisfaction with the service. The city’s current goal is to expand the number and diversity of digital services to gradually include other medical specialties and the option of scheduled consultations. These initiatives demonstrate the program’s commitment to equitable access, particularly for populations with limited connectivity or low digital literacy.

### Risk classification

Before Avança Saúde SP, patients with identical symptoms could have been admitted to different emergency care units in São Paulo and received different diagnoses, treatment and referral. The lack of pre-established criteria or protocols generated unequal data for the municipality, compromised the flow of care at the unit, put the patient’s health at risk and burdened the Secretariat’s budget with expenses for unnecessary examinations.

Enhancing the organization of medical and hospital care, as well as establishing clear processes for patient flow and referrals, became a key priority for Avança Saúde SP. To achieve this, Avança Saúde SP prioritized improving medical care organization and patient flow. The Health Department implemented the internationally recognized Manchester risk classification protocol, using a color-coded system to assess patient urgency, from urgent (red) to non-urgent (blue). The information is directly included in the E-Saúde SP platform and contains determinants to define the degree of urgency of the service.

The implementation of risk classification through the Manchester protocol began in 2020, during the coronavirus pandemic, in time to meet the demand for careful screening. Low-risk patients could be referred for care at basic health units or for teleconsultations, reducing overcrowding in emergency rooms and the risk of contamination and speeding up the hospitalization of severe cases.

The use of the Manchester protocol associated with E-Saúde SP reduced the average screening time in the emergency room from 6 to 2.5 min. The joint implementation of both systems has simplified the recording of the patient’s medical history, previously done on paper, and made care more agile, efficient and secure. Now available in 53 units, the protocol’s expansion included training >2000 primary care professionals—equivalent to ~20% of the municipality’s health workforce.

Combined with telemedicine, it enabled digital points of care in emergency units, improving patient flow. Low-complexity patients can opt for remote consultations at the unit, supported by on-site staff and technology.

### Results achieved with the management systems

Public health management in São Paulo lacked systems that offered an integrated view of contracts and supply flows. Outdated tools could not meet the growing need for automated data integration. This section describes investments in digital management systems designed to increase administrative efficiency, transparency and decision-making capacity. It details the implementation of the Integrated Partnership Control and Evaluation System (SICAP), the Administrative Contract Management System and the Centralized Supply Management system, all of which enable standardized workflows and real-time monitoring of contracts and inventory, and improved governance over municipal health resources.

### Integrated Partnership Control and Evaluation System

The Coordination of Partnerships and Contracting of Health Services (CPCs) of São Paulo is responsible for the assistance and financial control of a significant number of contracts and agreements for 76% (345) of the municipalities Primary Health Care units (UBS/AMA) in partnership with nine entities qualified as Social Organizations through Management Contracts and Agreements. The department is responsible for the Secretariat’s highest volume of financial transfers: ~R$800 million per month (USD 130.466 million) in payment transfers for >30 different contracts, involving >500 health units distributed among the 6 municipal regions.

Before Avança Saúde SP, CPCs used a system that functioned as a consolidator of dispersed, manually entered information. Data collection did not follow any automated capture strategies. The reliance on manual processes delayed responses to regional health office requests and increased the risk of data errors and inconsistencies. The situation was aggravated by the need to expedite emergency purchases during the pandemic.

The SICAP was contracted to reorganize workflows, enhancing security and transparency to the entire process of monitoring, controlling and evaluating service contracts with social organizations and outsourced companies in general. The new system offers viable conditions for internal and external control by inspection agencies, imposing a paradigm shift by creating a uniform data completion and validation flow across all territories. The system also allows the Secretariat to monitor indicators and measure results between different social organizations, evaluating criteria such as speed, transparency and efficiency in the execution of contracts and partnerships.

### Administrative contract management system

The administrative contracts division supports the entire Municipal Health Department, managing ~400 active contracts annually—including formalizations, amendments and guarantees. Despite its strategic role, the division operated entirely manually before Avança Saúde SP, with no system for integration, alerts or management support.

The sector’s demand increased even more during the pandemic, with numerous requests for contracts to be produced and signed under an emergency regime. New contractual models had to be created, with their own fields and drafts to be developed. Avança Saúde SP itself caused extra challenges for the team by contracting >100 projects and opening numerous bids to meet the program’s needs.

To streamline operations, a customizable platform was implemented in 2023, integrating spreadsheets and interoperating with other systems. It allows direct data entry and on-demand reporting, supported by a dedicated team from design to launch. The unified database brings more reliability, transparency and agility in accessing information. Only the contract team is authorized to edit the material, while professionals from different SMS management levels can view the information made available in the system and generate reports segmented by topics of interest.

### Procurement/contract systems and centralized supply management

The São Paulo Health Department manages the purchase and inventory of >6000 items, including supplies and medications, to serve almost 1000 health units spread over a 1509-km^2^ territory. Prior to Avança Saúde SP, each order involved the cross-analysis of data from >10 different platforms, monitored manually.

Without unified purchasing management or an alert system for possible inventory write-offs, São Paulo was facing problems with shortages. Patients who arrived at the basic health unit may have found that items available the previous day were no longer accessible. There was no inventory flow control to guarantee the advance purchase of items, an example of the technical-administrative challenges affecting healthcare and citizen health.

To coordinate demand, Avança Saúde SP envisaged the development and implementation of an integrated and interoperable procurement and contracting system using the main inventory, budget and logistics control tools available in the municipality. The challenge was to establish a system capable of controlling the flow of medicines and supplies from the Secretariat, anticipating inventory write-offs, average consumption per product and expiration dates, and entry and exit tickets for materials.

The new system integrates with several platforms, including Health Systems Management (GSS), which controls material flow in health units, and the municipality’s Budget and Financial System (SOF). This tool has led to more accurate and assertive purchasing decisions. Centralized orders generate equally unified deliveries, with single shipping per item, whether ordered for primary care or the hospital network. The payment flow can also be tracked end-to-end, from initial budget to billing. Finally, the tool can produce a report that covers all processing information—balances, coverage, purchase and payment, delivery and distribution, in a 360° management of the acquisition and inventory control of healthcare products in the municipality.

## DISCUSSION

The Avança Saúde SP program provides valuable lessons for strengthening a more resilient healthcare system. Investments in technology and management systems during the critical period of the COVID-19 pandemic enhanced the capacity to collect, analyze and act on real-time data**,** improving integrated responsiveness to emerging health challenges and enabling a coordinated, data-driven approach to care**.** These technological advancements played a pivotal role in ensuring that the primary health system remains adaptable and responsive to both anticipated and unforeseen circumstances, reinforcing its ability to withstand shocks while maintaining essential services.

The COVID-19 pandemic accelerated digital transformation in public health, highlighting vulnerabilities and the need for innovation. Digital tools like telecare and online health services, initially met with skepticism, became essential for care continuity during the crisis. Services such as E-Saúde SP, telecare consultations and the risk classification system enabled remote care, reduced virus exposure and improved efficiency. The integration of clinical data with the Vaccine Passport helped track immunization. These advancements laid the foundation for a more resilient, flexible and inclusive healthcare system.

Within the state, the scalability and resilience of Avança Saúde SP stemmed from institutional, governance and technical factors, reinforced by international support. The program combined IDB financing and technical cooperation with counterpart contributions from the São Paulo Municipality, aligning efforts to ensure implementation and sustainability. This framework, anchored in clear rules, indicators and cross-sectoral participation, helped the initiative endure administrative changes. The Municipal Health Department created a Project Coordination Unit (UCP) with administrative autonomy and specialized teams for planning, finance, procurement, technology and infrastructure, supported by an Institutional Coordination Committee of municipal secretariats and agencies. This structure allowed the UCP to design, execute and monitor the program while setting indicators to strengthen primary care and emergency networks. Strong political coordination and strategic leadership enabled the integration of interoperable digital platforms, providing a potentially replicable model for digital transformation and primary healthcare resilience in other urban settings.

Avança Saúde SP positioned São Paulo as the only municipality in Brazil to anticipate and exceed the minimum dataset (CMD) requirements established by the Brazilian Unified Health System, addressing the fragmentation of clinical-administrative data. Investments in the E-Saúde SP platform, along with telemedicine and risk classification systems, provided a comprehensive 360° view of primary healthcare, consolidating data from >26 million users into one of the largest health databases globally.

The E-Saúde SP platform and integrated risk classification system reduced emergency room wait times and redirected low-risk cases to basic health units or hybrid telemedicine offices. Patients and doctors now share responsibility for care management, as the platform enables alerts for test results**,** chronic disease monitoring**,** appointment scheduling and vaccination tracking**,** empowering patients to actively manage their health.

Digital tools such as My Health and the Glycemic Self-Monitoring Program have strengthened chronic disease management, improved prescription security and reduced repeat visits to health centers. This transformation streamlines primary care workflows, shortens queues and enhances access to continuous, data-driven care, particularly for patients with chronic conditions.

In 2024, SUS was named the best public service in São Paulo for the third consecutive year [16], praised for its digital tools like the Glycemic Self-Monitoring Program and the E-Saúde SP app. While the full impact of telecare is still being assessed, preliminary data highlight its effectiveness and acceptance.

The Avança Saúde SP’s emphasis on digital and technological tools focused on building data systems and intelligence with a focus on citizen empowerment has been fundamental to consolidating a strategic legacy, highlighting both strengths and critical gaps in São Paulo’s primary healthcare system. Moreover, the deployment of these digital solutions during the escalating health emergency supported a system-wide review aligned with the four phases of resilience—Shock & Alert, Responsiveness & Impact, Recovery & Learning and Preparedness & Prevention [17]—as illustrated in Table 1 above.

**Table 1:** Application of PAHO’s four phases of resilience to the Avança Saúde SP implementation process

**PAHO’s resilience framework**	**Key actions**	**Results achieved**	**Lessons learned**
Shock & Alert	Rapid adoption of digital platforms (E-Saúde SP repository and app), emergency telecare services, risk classification system during COVID-19	Enabled immediate identification of cases, prioritized high-risk patients, reduced ER overcrowding, facilitated vaccination campaigns	Early investment in interoperable digital tools allows health systems to respond quickly to shocks; proactive risk stratification enhances patient safety
Responsiveness & Impact	Telecare consultations, hybrid office model, home visits; data-driven management of supplies and contracts	1.7 million teleconsultations, reduced screening time from 6 to 2.5 min, improved contract management and supply allocation	Combining administrative and clinical systems strengthens real-time decision-making and equitable service delivery; digital health tools enhance operational efficiency
Recovery & Learning	Analysis of integrated data, refinement of workflows, evaluation of risk classification, continuous staff training	Integrated database of 26 million SUS cards, optimized patient flow, improved chronic disease management	Real-time data collection and feedback loops support learning and adaptation; empowers staff and patients to improve health outcomes
Preparedness & Prevention	Expansion of E-Saúde SP functionalities, standardized risk protocols, strengthened governance, community engagement via app	Increased access to care, patient empowerment, ongoing monitoring of chronic diseases and vaccination coverage	Building resilient PHC requires ongoing investment in interoperable systems, governance and community engagement to anticipate and prevent future crises

Over the 5 years of implementation, the Avança Saúde SP program often required the Shock & Alert and Responsiveness & Impact phases to occur simultaneously, reflecting the need for rapid adaptation and agility. This demanded high-level planning and decision-making skills to anticipate outcomes and facilitate a transition to the Recovery & Learning phase. São Paulo now emerges from the pandemic with a strengthened healthcare system, focused on leveraging lessons learned, and equipped with the tools and strategies needed to advance toward the Preparedness & Prevention phase—ensuring readiness to confront future health emergencies while promoting universal and equitable health coverage.

## METHODOLOGICAL CONSIDERATIONS

This case study draws on 13 interviews with São Paulo Health Department representatives and a review of secondary documents. While it provides an in-depth look at Avança Saúde SP and its digital transformation strategies, it reflects a single municipality and a limited set of informants, and quantitative data come mainly from administrative records. Despite these limitations, the study offers valuable insights into the design, implementation and potential scalability of integrated digital tools in primary healthcare, highlighting lessons for similar urban initiatives.

## STUDY FUNDING

This work was supported by the Inter-American Development Bank.

## APC FUNDING

This work was supported by the Inter-American Development Bank.

## Data Availability

The data underlying this article are available for use upon request and proper attribution to the authors.

## Notes

**Note**: This manuscript draws from a report by the Inter-American Development Bank, https://doi.org/10.18235/0013079
